# Retention Secured Nonlinear and Self‐Rectifying Analog Charge Trap Memristor for Energy‐Efficient Neuromorphic Hardware

**DOI:** 10.1002/advs.202205654

**Published:** 2022-11-27

**Authors:** Geunyoung Kim, Seoil Son, Hanchan Song, Jae Bum Jeon, Jiyun Lee, Woon Hyung Cheong, Shinhyun Choi, Kyung Min Kim

**Affiliations:** ^1^ Department of Materials Science and Engineering Korea Advanced Institute of Science and Technology (KAIST) Daejeon 34141 Republic of Korea; ^2^ Semiconductor Research & Development (SRD) Samsung Electronics Hwaseong 18448 Republic of Korea; ^3^ The School of Electrical Engineering Korea Advanced Institute of Science and Technology (KAIST) Daejeon 34141 Republic of Korea

**Keywords:** analog, charge‐trap, memristors, neuromorphic, self‐rectifying

## Abstract

A memristive crossbar array (MCA) is an ideal platform for emerging memory and neuromorphic hardware due to its high bitwise density capability. A charge trap memristor (CTM) is an attractive candidate for the memristor cell of the MCA, because the embodied rectifying characteristic frees it from the sneak current issue. Although the potential of the CTM devices has been suggested, their practical viability needs to be further proved. Here, a Pt/Ta_2_O_5_/Nb_2_O_5‐_ *_x_*/Al_2_O_3‐_ *_y_*/Ti CTM stack exhibiting high retention and array‐level uniformity is proposed, allowing a highly reliable selector‐less MCA. It shows high self‐rectifying and nonlinear current‐voltage characteristics below 1 µA of programming current with a continuous analog switching behavior. Also, its retention is longer than 10^5^ s at 150 °C, suggesting the device is highly stable for non‐volatile analog applications. A plausible band diagram model is proposed based on the electronic spectroscopy results and conduction mechanism analysis. The self‐rectifying and nonlinear characteristics allow reducing the on‐chip training energy consumption by 71% for the MNIST dataset training task with an optimized programming scheme.

## Graphical abstract

Pt/Ta_2_O_5_/Nb_2_O_5‐_ *_x_*/Al_2_O_3‐_ *_y_*/Ti charge trap memristor exhibiting high retention and array‐level uniformity with self‐rectifying and low operation range is proposed. A novel operation model based on electronic spectroscopy and conduction mechanism analysis is suggested. With the self‐rectifying and nonlinear characteristics, energy consumption decreases by 71% with an optimized programming scheme.

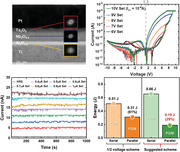

## 1 Introduction

A memristive crossbar array (MCA) attracts intensive attention due to its various emerging applications, such as high‐density storage class memory or artificial synapse network for neuromorphic computing.^[^ ^1^ ^]^ The significant advantage of the MCA is its high‐density capability; the cell requires only 4*F* ^2^ (*F* = minimum feature size) area, and it can be easily stackable either vertically or horizontally, which can multiply its areal density. Furthermore, its multi‐level programming capability can provide even higher bit‐storage density. Despite such a high potential, the MCA may suffer from a sneak current problem in both reading and programming due to the missing of the cell transistor. Inserting a two‐terminal selector in series with the memristor cell can be a viable solution,^[^ ^2^ ^]^ but it requires a significant burden in material development and integration processing.

In this regard, charge trap memristors (CTM) are promising memristive systems due to their inherent forming‐free and self‐rectifying characteristics.^[^ ^3^, ^4^, ^5^, ^6^, ^7^, ^8^, ^9^, ^10^, ^11^ ^]^ Also, they show analog conductance change characteristics at low operation current range, enabling high‐density MCA applications.^[^ ^12^, ^13^ ^]^ The CTM mechanism is identical to the charge‐trap flash (CTF) memory, in which reversible charge trapping and de‐trapping to the charge trap layer alter the threshold voltage of the transistor.^[^ ^14^, ^15^, ^16^ ^]^ Unlike the three‐terminal CTF, the two‐terminal CTM directly reads the conductance change through the charge trap layer, allowing higher density and simpler operation.^[^ ^17^, ^18^, ^19^ ^]^ However, as the programming and reading operations share identical terminals, there is a contradiction between the stability of the trapped charges (i.e., retention) and the reliability of the programming process (i.e., writing or erasing).^[^ ^20^ ^]^ The higher stability of the trapped charges can be achieved by reducing the tunneling probability of the trapped charges by forming thicker oxides. However, they make the charge trapping and de‐trapping process difficult, increasing the programming voltage or time. To achieve both high stability and programming reliability, it is crucial to understand the charge trapping and de‐trapping processes, as well as developing an optimized device stack.

Meanwhile, nonlinear current–voltage (*I*–*V*) characteristics (curvature of the *I*–*V* curve at the forward bias) are also crucial for high‐density memory applications in the MCA. It was demonstrated that inserting a nonlinear selector device in series with the self‐rectifying CTM could reduce the total power consumption by suppressing the unnecessary power consumption from half‐selected cells.^[^ ^7^ ^]^ If the nonlinear characteristic is embedded inherently, larger array integration would be possible, and the practicality of the CTM‐based MCA could be further secured.

In this work, we propose a Pt/Ta_2_O_5_/Nb_2_O_5‐_ *_x_*/Al_2_O_3‐_ *_y_*/Ti (PTNAT) CTM device exhibiting > 5 × 10^4^ of self‐rectifying ratio, < 1 µA of programming current, > 10^5^ cycles of endurance, and > 2 × 10^5^ seconds of retention at 150 °C, which is suitable for the high‐density memory applications. We propose a plausible CTM mechanism model accounting for the retention‐secured characteristics based on systematic experiments and electronic spectroscopy analysis results. Also, we show its high uniformity from a 32 × 32 array device and demonstrate its neuromorphic device operation. With an optimized weight update biasing scheme utilizing the self‐rectifying and nonlinear characteristics, the system can achieve high accuracy (≈91%) at the MNIST dataset recognition challenge with only 29% of energy consumption compared to the conventional programming scheme.

## 2 Results and Discussion

### 2.1 Electrical Characteristics of the Pt/Ta_2_O_5_/Nb_2_O_5‐_ *_x_*/Al_2_O_3‐_ *_y_*/Ti Charge Trap Memristor Device

**Figure 1:**
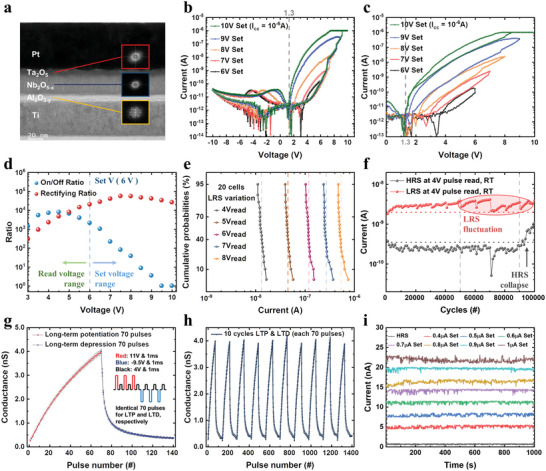
Pt/Ta_2_O_5_/Nb_2_O_5‐_ *_x_*/Al_2_O_3‐_ *_y_*/Ti (PTNAT) cell characteristics. a) The cross‐section transmission electron microscopy (TEM) image of the device and fast‐Fourier transform (FFT) image. b) The resistance switching *I*–*V* curves of the device with 1 µA compliance current (*I*_CC_) measured at different positive voltage sweeps (6–10 V) and −10 V fixed voltage sweeps. c) *I*–*V* curves with continuously increasing voltage sweeps (6–10 V) without reset. d) The on/off ratio and the rectifying ratio as a function of the applied voltages. e) The cumulative probabilities of the current levels of LRS in the positive voltage region, which were obtained from the first voltage sweeps in 20 cells. f) The endurance properties up to 10^5^ cycles read at 4 V at room temperature. g) The average and standard deviation of both LTP and LTD for 70 pulses during ten cycles. h) 10 cycles of LTP and LTD operation of the device. i) The retention characteristics of 3‐bit intermediate states at 125 °C at 2 V.

**Figure** **1**a shows a cross‐sectional transmission electron microscopy (TEM) image of the PTNAT CTM structure, where the thicknesses are ≈10 nm for Ta_2_O_5_, ≈28 nm for Nb_2_O_5‐_ *_x_*, and ≈8 nm for Al_2_O_3‐_ *_y_*. All oxides are amorphous, confirmed by the fast‐Fourier transform of the TEM (insets in Figure 1a) and XRD measurements (see Figure S1, Supporting Information, for the XRD results). Figure 1b shows analog *I*–*V* characteristics of 25 µm^2^‐area device by various positive set voltages from 6 to 10 V, followed by a fixed ‐10 V reset voltage. The device is electroforming‐free, and its pristine state is the high resistance state (HRS). Any intermediate resistance states between the HRS and the low resistance state (LRS) can be attained by either changing a compliance current (*I*_cc_) or the maximum set voltage. Figure 1c shows analog *I*–*V* curves, where the applied voltage was consecutively increased from 6 to 10 V without the reset process. The analog switching characteristic is attributed to a continuous charge trapping into the defect states of the Nb_2_O_5‐_ *_x_* layer. As the amount of trapped charges increases, they form a higher internal negative field, lowering the Schottky barrier height (SBH) at the Ta_2_O_5_/Nb_2_O_5‐_ *_x_* interface. Also, the *I*–*V* curves show a flat band voltage (*V*_fb_) at ≈1.3 V (gray dashed line). This can increase the nonlinearity in the forward bias, making it more beneficial for the energy‐efficient array operation. More details for the switching modeling and the array demonstration will be discussed in the following sections. Figure 1d plots the on/off ratio (= *I*_LRS_(V) / *I*_HRS_(V)) and the rectifying current ratio (= *I*_LRS_(V) / *I*_LRS_(‐V)) as a function of the voltage, obtained from 10 V/−10 V DC *I*–*V* sweep curve of Figure 1b. The maximum on/off ratio is ≈8 × 10^3^ at 4.5 V. In the high voltages (>6 V, the set threshold voltage), the device set‐switched gradually, so the on/off ratio decreased accordingly. At 10 V, the device set‐switched completely to the LRS, so the on/off ratio collapsed to 1. The maximum rectifying current ratio is ≈5.7 × 10^4^ at 7.5 V, and the rectifying ratio exceeds 10^4^ over a wide voltage range exceeding ≈5.0 V at the LRS, suggesting the device may efficiently suppress the sneak currents during both reading and programming. To minimize the sneak currents, get a high on/off window, and have sufficient voltage margin with the set voltage (≈6 V), the optimal read voltage could be around 4 to 5 V. Figure 1e shows cumulative probability of the LRS current levels at various read voltages from 4 to 8 V. The dashed lines are the average current values obtained from the first voltage sweeps of 20 cells, confirming high cell‐to‐cell uniformity. Note that the device is electroforming‐free, which can realize higher uniformity than the electroformed devices. Figure 1f shows a cycling endurance up to 10^5^, where the set and reset pulse conditions were 13 V for 10 ms and −13 V for 10 ms, and the reading pulse was 4 V for 1 ms. This endurance is comparable with the endurance of the commercialized storage (compare NAND flash guarantees about 10^5^ endurance), suggesting it is sufficient for non‐volatile memory applications. After ≈5 × 10^4^ cycles, the LRS current was unstable, which is associated with the electron trapping at the Al_2_O_3‐_ *_y_* tunneling oxide. In the NAND flash, it is well‐known that the program/erase (P/E) efficiency can be deteriorated over P/E cycles due to the oxide aging and unwanted additional electron trapping.^[^ ^21^, ^22^ ^]^ Similarly, the tunneling oxide may be aged over cycling, leading to unintended charge trapping and the LRS fluctuation. After 10^5^ cycles, the HRS collapsed to the LRS, meaning the trapped charges could not escape from the traps. This is associated with the deterioration of the Nb_2_O_5‐_ *_x_* deep trap sites, which form permanent fixed charges. Figure 1g shows long‐term potentiation (LTP) and long‐term depression (LTD) behaviors for the artificial synapse application. The inset shows the pulse conditions for LTP and LTD. The conductance was read by 4 V to prevent the unwanted conductance change during reading. Potentiation and depression pulse amplitudes were set to 11 and −9.5 V, which could achieve the full memory window. These voltages can readily offer a high rectifying ratio as shown in Figure 1d, so it can be used in the array operation, which is discussed later. Figure 1h shows 10 LTP and LTD cycles, confirming a high cycling uniformity. Figure 1i shows retention characteristics of selected eight conductance states at 125 °C (read at 2 V), verifying high stability up to 1000 s at the elaborated temperature except for some white noise from the measurement system.

### 2.2 Study on the Retention Characteristics of the Pt/Ta_2_O_5_/Nb_2_O_5‐_ *_x_*/Al_2_O_3‐_ *_y_*/Ti Device

**Figure 2:**
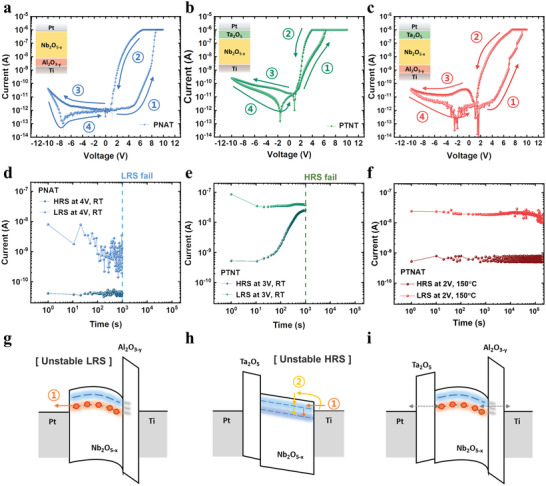
Retention tendency comparison with 2‐layer and 3‐layer devices. The resistance switching *I*–*V* curves of the a) Pt/Nb_2_O_5‐_ *_x_*/Al_2_O_3‐_ *_y_*/Ti (PNAT), b) Pt/Ta_2_O_5_/Nb_2_O_5‐_ *_x_*/Ti (PTNT), and c) Pt/Ta_2_O_5_/Nb_2_O_5‐_ *_x_*/Al_2_O_3‐_ *_y_*/Ti (PTNAT) device. Each *I*–*V* curve was measured with a 10 V positive voltage sweep and a −10 V negative voltage sweep with a 10^−6^ A compliance current. The retention characteristics of d) PNAT (4 V at room temperature (RT)), e) PTNT (3 V at RT), and f) PTNAT (2 V at 150 °C). Schematic energy band diagram models of g) PNAT, h) PTNT, and i) PTNAT devices.

To investigate the high retention of the PTNAT device, we investigated *I*–*V* curves and retention characteristics of Pt/Nb_2_O_5‐_ *_x_*/Ti (PNT), Pt/Nb_2_O_5‐_ *_x_*/Al_2_O_3‐_ *_y_*/Ti (PNAT), Pt/Ta_2_O_5_/Nb_2_O_5‐_ *_x_*/Ti (PTNT), and PTNAT devices. The PNT device showed a narrow on/off ratio due to a high leakage current with a low set switching voltage of 3 V. (see Figure S2a, Supporting Information, for the PNT device *I*–*V* data.) Also, the device returned to the HRS shortly after the set switching, meaning the trapped charges were de‐trapped quickly as soon as the electric field was removed. When the Al_2_O_3‐*y*_ layer was inserted at the bottom (i.e., PNAT device), the on/off ratio was drastically improved by suppressing the HRS current and the rectifying characteristic was revealed, as shown in **Figure** **2**a. Compared with the PNAT device, when the Ta_2_O_5_ layer was inserted underneath the Pt (PTNT device), the HRS was still leaky, as shown in Figure 2b, meaning the upper Ta_2_O_5_ layer was not suppressing the leakage current. Figure 2c shows the *I*–*V* curve of the PTNAT device with both the upper Ta_2_O_5_ and the lower Al_2_O_3‐_ *_y_* layers.

The retention characteristics of the three devices were also distinguishable. In Figure 2d, the PNAT device showed improved stability than the PNT device. However, the LRS collapsed to the HRS, so the on/off ratio was reduced from ≈200 to ≈10 after ≈1000 s. It means the trapped charges are still unstable, so they may escape from the trap sites to the Pt electrode. Meanwhile, it suggests that the Al_2_O_3‐_ *_y_* layer may stabilize the HRS. In the PTNT device, the retention failure trend was different compared to the PNT and PNAT devices; the HRS collapsed to the LRS, as shown in Figure 2e. It suggests that the upper Ta_2_O_5_ layer may suppress the de‐trapping of charges and stabilize the LRS, although the HRS was unstable. Eventually, the PTNAT device showed stable LRS and HRS even at the harsh temperature, as shown in Figure 2f (150 °C, read at 2 V).

The retention tendency could be understood with the schematic energy band diagram of each device. For the band structure modeling, reflection energy loss spectroscopy (REELS) and ultraviolet photoelectron spectroscopy (UPS) analysis were performed. (see Figure S3, Supporting Information, for the REELS and UPS results of Al_2_O_3‐_ *_y_*, Nb_2_O_5‐_ *_x_*, and Ta_2_O_5_ layers.) From the analysis results, the bandgap (*E*_g_) and the electron affinity (*χ*) values of each dielectric layer were obtained. The estimated *E*_g_ values from the threshold energy of the REELS were 6.6 eV (Al_2_O_3‐_ *_y_*), 4.2 eV (Nb_2_O_5‐_ *_x_*), and 4.5 eV (Ta_2_O_5_), respectively. The UPS analysis gave the *χ* of each layer to 2.29 eV (Al_2_O_3‐_ *_y_*), 4.03 eV (Nb_2_O_5‐_ *_x_*), and 3.44 eV (Ta_2_O_5_).

From the analysis results, the band diagram of each device can be suggested, as shown in Figure 2g–i. In all devices, two trap levels are present; a deep trap level around ≈1.1 eV below the conduction band and a shallow trap level around ≈0.22 eV below the conduction band, which are attributed to the oxygen vacancies of the amorphous Nb_2_O_5‐_ *_x_* layer.^[^ ^23^ ^]^ The Pt/Ta_2_O_5_/Al_2_O_3‐_ *_y_*/Ti (PTAT) device missing the Nb_2_O_5‐_ *_x_* layer did not show memory operation, confirming that the Nb_2_O_5‐_ *_x_* layer is responsible for the charge trapping. (see Figure S2b, Supporting Information, for the PTAT device *I*–*V* data). Among the two trap levels, the deep trap level is responsible for the charge trap‐based memory operation. The shallow trap level acts as a stepping‐stone‐like energy state, affecting the charge trapping and de‐trapping process, which is elaborated in the modeling section.

The retention failure mechanisms can be suggested in the given band diagram. In the PNAT device (Figure 2g), the trapped charges can easily escape from the traps toward the Pt electrode as there is no blocking barrier at the top interface. The de‐trapping is possible via direct tunneling from the traps to the Pt electrode. In the PTNT device (Figure 2h), the trap sites are spontaneously trapped over time due to the low activation energy for the electron tunneling process from the Ti bottom electrode to the Nb_2_O_5‐_ *_x_* layer, while the upper Ta_2_O_5_ layer suppresses the de‐trapping process. Therefore, an LRS failure was observed. In addition, the shallow traps at the Nb_2_O_5‐_ *_x_*/Ti interface generated by the oxygen scavenging effect of the Ti electrode may help the charge trapping process via band‐to‐trap direct tunneling. Lastly, in the PTNAT device (Figure 2i), both Ta_2_O_5_ and Al_2_O_3‐_ *_y_* dielectric layers stabilize the trapped charges and suppress spontaneous trapping even at high temperatures.

### 2.3 Conduction Mechanism Analysis and Band Diagram Modeling of Pt/Ta_2_O_5_/Nb_2_O_5‐_ *_x_*/Al_2_O_3‐_ *_y_*/Ti Device

**Figure 3:**
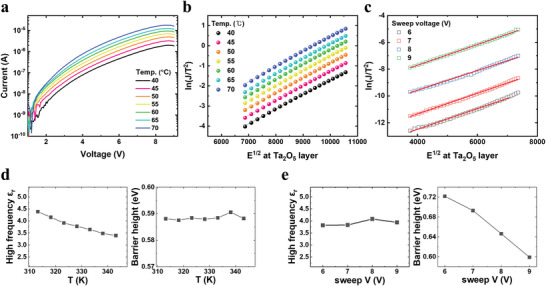
Conduction mechanism fitting of PTNAT device. a) *I*–*V* curves of the LRS measured at temperatures ranging from 40 to 70 °C. b) Schottky emission form (ln(*J*/*T* ^2^) vs *E* ^1/2^) plot for the voltages ranging from 3.5 to 6.5 V in LRS with the calculated Ta_2_O_5_ layer partial electric field. c) Analog LRS states Schottky emission fitting in +4.5 V read voltage and room temperature condition after +6 to +9 V different sweep voltages. d) High‐frequency permittivity (*ε*_op_) and Schottky barrier height (*Φ*_B_) extracted at each temperature condition *I*–*V* curves. e) *ε*_op_ and *Φ*_B_ values extracted at each analog state. *Φ*_B_ decreased with a more programmed analog state (higher conductance state).

Conduction mechanisms of the PTNAT device were examined to confirm the proposed band diagram model and understand the detailed switching mechanism. **Figure** **3**a shows the LRS *I*–*V* curves measured at temperatures ranging from 40 to 70 °C. The results revealed that the LRS is highly temperature‐dependent, suggesting that the conduction is associated with the thermionic conduction mechanism. For more detailed analysis, the temperature‐dependent *I*–*V* curves at a high bias region (3.5–6.5 V) were plotted to the Schottky emission equation (ln(*J*/*T* ^2^) vs *E* ^1/2^), as shown in Figure 3b. In addition, multiple LRS curves programmed by different voltages from +6 to +9 V at fixed room temperature were plotted with the Schottky emission equation, as shown in Figure 3c. Both plots confirmed that the Schottky emission dominates the conduction of the device.^[^ ^24^ ^]^ From the fitting, the optical dielectric constant (*ε*_op_) and SBH (*Φ*_B_) of the conduction‐limiting interface could be extracted at each temperature and each conductance state, as shown in Figure 3d and Figure 3e, respectively. For the fitting, the local electric field on each layer was calculated using the thickness and dielectric constant of the oxide layers. The thicknesses were 9.7 nm of Ta_2_O_5_, 27.7 nm of Nb_2_O_5‐_ *_x_*, and 8.3 nm of Al_2_O_3‐_ *_y_* from the TEM image in Figure 1a. The dielectric constants were 24 for Ta_2_O_5_,^[^ ^25^ ^]^ 45 for Nb_2_O_5‐_ *_x_*,^[^ ^26^ ^]^ and 9 for Al_2_O_3‐_ *_y_*,^[^ ^27^ ^]^ which were taken from literature.

After investigating all possible cases, we could conclude that the Schottky barrier at Ta_2_O_5_/Nb_2_O_5‐_ *_x_* interface limited the overall conduction (see Figure S4, Supporting Information, for the conduction mechanism fitting results limited by other layers.) The estimated *ε*_op_ values ranged from 4.4 to 3.4 as the temperature increased from 40 to 70 °C, which coincided with the reference value (*ε*_op_ ≈ 4) of Ta_2_O_5_ calculated by the square of its refractive index (*n* ≈ 2). In addition, the estimated *Φ*_B_ ranged from 0.588 to 0.591 eV at 40–70 °C, suggesting the band offset at Ta_2_O_5_/Nb_2_O_5‐_ *_x_* interface. (The effective electron mass condition (*m*_e_ ≈ 0.3 *m*_0_) of Ta_2_O_5_ was used for the *Φ*_B_ calculation.^[^ ^28^ ^]^) This band offset also coincides with the *χ* difference of the Ta_2_O_5_/Nb_2_O_5‐_ *_x_* interface measured from the UPS analysis, 4.03 eV (Nb_2_O_5‐_ *_x_*), and 3.44 eV (Ta_2_O_5_). Similarly, the *ε*_op_ and *Φ*_B_ were also calculated from multiple conductance states. The estimated *ε*_op_ was 3.82–4.08, consistent with the temperature‐dependent fitting results in Figure 3d. Interestingly, the *Φ*_B_ were decreased from 0.72 eV (lower conductance state) to 0.60 eV (higher conductance state) as the set voltage increased, suggesting the *Φ*_B_ decreased by further charge trapping. Such *Φ*_B_ modulation behavior with respect to the charge trapping process can be plausibly explained by the following switching model.

**Figure 4:**
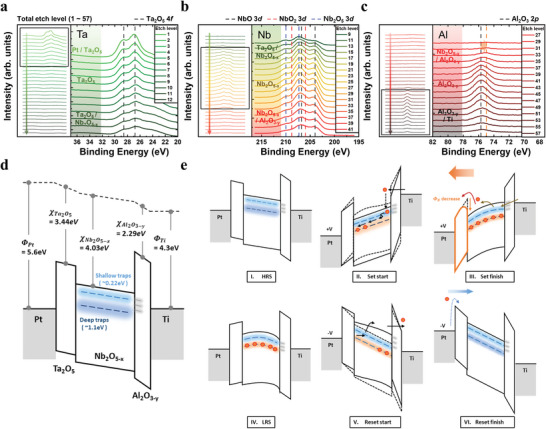
PTNAT XPS depth profile results and resistance switching mechanism of the device. Depth profiling results of a) Ta 4*f*, b) Nb 3*d*, and c) Al 2*p* core levels of the Ta_2_O_5_(10 nm)/Nb_2_O_5‐_ *_x_* (28 nm)/Al_2_O_3‐_ *_y_* (8 nm)/Ti sample. The left panels show the raw XPS depth profile data. The right panels enlarge the square region. d) The schematic energy band diagram of the device for zero bias condition. Shallow and deep trap levels exist in the non‐stoichiometric Nb_2_O_5‐_ *_x_* layer. e) The illustration of the resistance switching process with charge trapping and de‐trapping. In the HRS, deep traps are empty, and conduction is not fluent with the high band offset at the Ta_2_O_5_ interface (I). When a positive set bias is applied on the Pt electrode, the electrons can be trapped in the Nb_2_O_5‐_ *_x_* deep trap sites (II). With the induced negative space charge, the partial electric field across the Ta_2_O_5_ layer increased (III), and LRS is achieved by effective Schottky barrier height lowering (IV). When a negative bias is applied (V, VI), the trapped electrons can be released to the Ti electrode, and the HRS is obtained.

**Figure** **4**a–c shows the XPS depth profile results of the Ta 4*f*, Nb 3*d*, and Al 2*p* core levels at each dielectric layer of the PTNAT device. The left panels show the raw XPS depth profile data (etch level from 1 to 57) to navigate the etch levels of interest (right panels) for each figure. In Figure 4a, the binding energy of two peaks have coincided with the reference Ta_2_O_5_ phase peaks (black dashed lines at 26.8 eV for Ta 4*f*_7/2_ and 28.6 eV for Ta 4*f*_5/2_).^[^ ^29^ ^]^ The core levels showed no specific changes with the etch level increase, meaning a stoichiometric Ta_2_O_5_ layer was formed by the plasma‐enhanced atomic layer deposition (PEALD) process. Figure 4b shows Nb 3*d* core levels with the reference peak positions are as follows: Nb^5+^ (Nb_2_O_5_) peaks at 210.0 and 207.3 eV (blue dashed line), Nb^4+^ (NbO_2_) peaks at 208.8 and 206.0 eV (red dashed line), and Nb^2+^ (NbO) peaks at 206.8 and 204 eV (black dashed line).^[^ ^30^ ^]^ The XPS results of the Nb_2_O_5‐_ *_x_* layer suggested complicated composition of various sub‐oxide phases, meaning a significant amount of oxygen vacancies exist in the Nb_2_O_5‐_ *_x_* charge trap layer. (see Figure S5, Supporting Information, for the Nb 3*d* XPS spectra deconvolution results of the PTNAT device.) At the Nb_2_O_5‐_ *_x_*/Al_2_O_3‐_ *_y_* interface, the peaks shifted toward the lower binding energy slightly, meaning the higher oxygen vacancies at the interface. Moreover, Al 2*p* peaks also showed lower binding energy shifting (orange dashed line) characteristics at the Nb_2_O_5‐_ *_x_*/Al_2_O_3‐_ *_y_* interface compared with the bulk region (black dashed line),^[^ ^31^ ^]^ as shown in Figure 4c. Both indicate that a high concentration of oxygen vacancies is formed at the Nb_2_O_5‐_ *_x_*/Al_2_O_3‐_ *_y_* interface. This may be originated from the sputtering damage during the Nb_2_O_5‐_ *_x_* deposition kicking off the oxygens from the underlying Al_2_O_3‐_ *_y_* layer. Eventually, high‐density trap levels were formed at the interface helping the trap‐assisted tunneling process.

Based on the conduction mechanism study and REELS, UPS, and XPS results, we could establish a detailed switching model of the PTNAT device. Figure 4d shows an energy band diagram of the PTNAT device. The work functions (*Φ*) of the electrodes were assumed to 5.6 eV for Pt top electrode and 4.3 eV for Ti bottom electrode. The bandgap (*E*_g_) and the electron affinity (*χ*) values were estimated from REELS and UPS results in Figure S3, Supporting Information, which are 4.5 and 3.44 eV for Ta_2_O_5_; 4.2 and 4.03 eV for Nb_2_O_5‐_ *_x_*; and 6.6 and 2.29 eV for Al_2_O_3‐_ *_y_*, respectively. As mentioned above, the Nb_2_O_5‐_ *_x_* charge trap layer contains two trap levels (≈0.22 eV and ≈1.1 eV below the conduction band) associated with the oxygen vacancies. The deep trap level is responsible for the charge trapping. The shallow trap levels assist fluent carrier injection via Poole‐Frenkel (P‐F) emission.^[^ ^32^ ^]^ Also, in the Al_2_O_3‐_ *_y_* layer, oxygen vacancies were observed at the Nb_2_O_5‐_ *_x_*/Al_2_O_3‐_ *_y_* interface. These defects could enhance the band‐to‐trap tunneling through the Al_2_O_3‐_ *_y_* layer, allowing the Al_2_O_3‐_ *_y_* to act as a tunneling layer despite the high band offset at the Al_2_O_3‐_ *_y_*/Ti interface.

With the proposed band diagram, the analog charge trapping associated resistance switching characteristics can be well understood. Figure 4e shows schematic band diagrams during the set and reset process. In the pristine HRS state (panel i), the deep traps are empty, and the conduction is not fluent due to the high SBH at the Ta_2_O_5_/Nb_2_O_5‐_ *_x_* interface. When a positive set bias (>6 V) is applied to the Pt top electrode, the injected electrons start to transit to the deep traps and act as negative space charges. They form an internal electric field and pull up the conduction band, resulting in two changes to the programming process; first, it lowers the SBH at the Ta_2_O_5_/Nb_2_O_5‐_ *_x_* interface, making the conduction more fluent. Second, it also pulls up the shallow trap levels relative to the Fermi level of the Ti electrode and suppresses additional charge trapping. Therefore, the set switching process stops at the given set switching voltage, making an analog conductance change possible.

Once the space charges are stored, the SBH at the Ta_2_O_5_/Nb_2_O_5‐_ *_x_* interface can be decreased by an image force‐associated Schottky barrier lowering effect.^[^ ^33^ ^]^ The amount of barrier lowering depends on the applied partial electric field across the dielectric film, which can be given as

(1) $$\Delta \phi \left(E\right)=\sqrt{e^{3}E/4\pi \varepsilon_{s}}+e\alpha E^{2}$$

where *α* is a material constant given by

(2) $$\alpha =e\hbar^{2}/24m^{*}{\left(k_{B}T\right)}^{2}$$

*ε*_s_ is a permittivity, *h* is the Plank constant, and m* is the effective electron mass. Thus, as more charges are trapped, the lower SBH is formed. These trapped charges are stable due to the high band offset at the Ta_2_O_5_/Nb_2_O_5‐_ *_x_* and Nb_2_O_5‐_ *_x_*/Al_2_O_3‐_ *_y_* interfaces, as shown in Figure 2f. At a sufficient negative bias for reset, the trapped charges are de‐trapped by the reverse sequence of the trapping process from deep traps via the shallow traps to the electrode.

### 2.4 Demonstration of Energy‐Efficient Neuromorphic Computing

**Figure 5:**
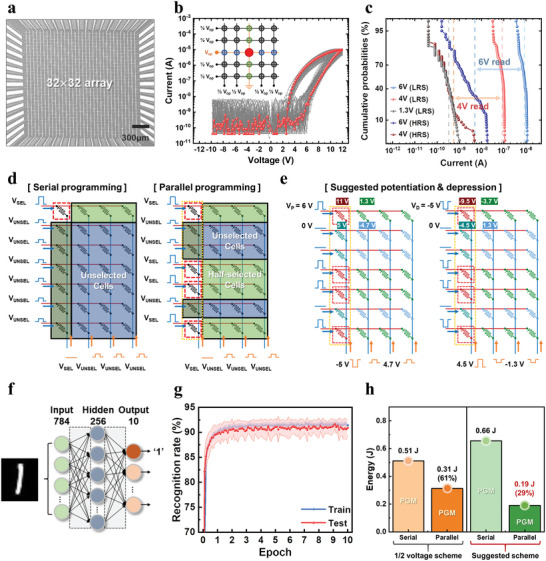
32 × 32 MCA integration and energy‐efficient neuromorphic computing demonstration. a) A top‐view SEM image of the 32 × 32 MCA. b) *I*–*V* curves of randomly chosen 80 cells with the half voltage scheme. The inset shows the half voltage array measurement scheme. c) The cumulative probabilities of the current levels at the LRS (read voltages of 1.3, 4, and 6 V), and the HRS (read voltages of 4 and 6 V). d) The serial (left) and parallel (right) programming schemes for weight update. e) Suggested potentiation and depression scheme for parallel programming. f) The double‐layer perceptron neural network used for the MNIST dataset training simulation. It consists of 784 input neurons, 256 hidden neurons, and 10 output neurons. g) The MNIST data recognition rate by training epoch considering the experimental synaptic characteristics of the CTM device. The final accuracy was about 91%. h) Estimated energy required for training by serial and parallel programming in half voltage scheme and those in the suggested scheme.

We fabricated a 32 × 32 MCA embedding the PTNAT cell, and examined an energy‐efficient on‐chip training method to get the maximum benefits of the device. **Figure** **5**a shows a top‐view image of the integrated device taken by scanning electron microscope, where the line width of the crossbar is ≈5 µm. Figure 5b shows the *I*–*V* curves of randomly selected 80 cells (gray), and one representative curve (red), confirming high array‐level uniformity. The current sensing limit of the array testing platform was about ≈10^−8^ A, so the lower current levels were not detectable. Nevertheless, the read currents at 4 V and 6 V were distinguishable with high uniformity, as shown in Figure 5c.

At on‐chip training, the energy consumption would be high if all cells in the array were updated individually. The self‐rectifying and nonlinear behaviors of the device can offer a way of energy‐efficient programming. It is reported that parallel programming could reduce energy consumption compared to the conventional serial programming method by decreasing the total number of programming events and energy consumption per event.^[^ ^34^ ^]^ Figure 5d compares the conventional serial programming and proposed parallel programming schemes. In parallel programming, the portion of the half‐selected cells is considerable, so managing both unselected and half‐selected currents is crucial. In the proposed PTNAT device, both the unselected cell and the half‐selected cell currents can be suppressed due to self‐rectifying and nonlinear behaviors, allowing the parallel programming scheme to be viable. Figure 5e illustrates parallel programming biasing schemes of potentiation (left panel) and depression (right panel) operations. For potentiation, the selected word and bit lines are biased to 6 and −5 V, respectively, offering a net 11 V potential for programming, which is the identical potentiation condition of Figure 1g. Meanwhile, the unselected word and bit lines are biased to 0 and 4.7 V, resulting in 1.3 V of the half‐selected cell voltage and −4.7 V of the unselected cell voltage, respectively. As shown in Figure 1b, 1.3 V corresponds to the flat band voltage, allowing a minimum leakage current at the half‐selected cells. Similarly, for depression, the selected word and bit lines are biased to −5 and 4.5 V, while the unselected bit and word lines are biased to 0 and −1.3 V, respectively, making the half‐selected cell voltage to −3.7 or −4.5 V, and the unselected cell voltage to 1.3 V.

The energy consumption of the CTM array as neuromorphic hardware was examined at a software‐based emulator embodying the CTM properties for the synapse device. The emulator can virtually expand the array size to the practical level and calculate the energy consumption accurately. The rationality of the methodology has been proven by multiple groups.^[^ ^35^, ^36^, ^37^ ^]^ For the energy‐efficiency examination, a fully‐connected double‐layer perceptron (DLP) neural network (constituting 784 input neurons, 256 hidden neurons, and 10 output neurons) was employed, and the MNIST dataset training task was performed, as illustrated in Figure 5f. The DLP was trained by the backpropagation algorithm, and the weight change was obtained by the stochastic gradient descent^[^ ^38^ ^]^ optimization method. The network was trained with 60 000 training datasets using a batch size of 50 for 10 epochs. For the simulation, a bi‐directional weight update system was adopted, where positive and negative weight values were represented by two CTM cells,^[^ ^35^, ^37^ ^]^ making a total of 407 060 synaptic cells in the network. For the weight update, oneshot update method using the analog characteristic of Figure 1g as a look‐up table was used (see Figure S6, Supporting Information, for the details of the neural network simulation and Figure S7, Supporting Information, for the fitting parameters). At each weight update, each cell consumes energy corresponding to its biasing condition, which can be expressed as follows:

(3) $$E_{\mathrm{tot}\left(\mathrm{pgm}\right)}=E_{\mathrm{selected}}+E_{\mathrm{half}-\mathrm{selected}}+E_{\mathrm{unselected}}$$

(4) $$E_{\mathrm{selected}}=\sum_{\mathrm{cell}}\frac{\left(G_{ij}^{\mathrm{ini}}+G_{ij}^{\mathrm{fin}}\right)}{2}{\left(V_{\mathrm{pgm}}\right)}^{2}n_{\mathrm{pulse}}t_{\mathrm{pulse}}$$

(5) $$E_{\mathrm{half}-\mathrm{selected}}=\sum_{\mathrm{cell}}G_{ij}^{\mathrm{ini}}{\left(V_{\mathrm{half}-\mathrm{selected}}\right)}^{2}n_{\mathrm{pulse}}t_{\mathrm{pulse}}$$

(6) $$E_{\mathrm{unselected}}=\sum_{\mathrm{cell}}G_{ij}^{\mathrm{ini}}{\left(V_{\mathrm{unselected}}\right)}^{2}n_{\mathrm{pulse}}t_{\mathrm{pulse}}$$

where $G_{ij}^{\mathrm{ini}}$ and $G_{ij}^{\mathrm{fin}}$are conductance values before and after weight update, *V*_pgm_, *V*_half − selected_, and *V*_unselected_ are node voltages of the cells, *n*_pulse_ is the number of potentiation pulses, and *t*_pulse_ is the pulse time. Figure 5g shows the estimated MNIST recognition rate of the device for the training and test dataset for 10 epochs of simulation. Our simulation showed ≈91% accuracy, which is lower than the ideal network accuracy of ≈97% possible in the given DLP network. This is due to the non‐ideal linearity characteristics and the conductance variation of the analog states.^[^ ^39^ ^]^ Li, C. et al. proposed that almost ideal accuracy can be achieved on a 1T1R memristive array, suggesting that linearity improvement is crucial for better accuracy.^[^ ^40^ ^]^ Although our device is not ideal yet, the estimated accuracy is comparable to other hardware‐based simulation performances ranging from 70% to 94%.^[^ ^41^, ^42^, ^43^, ^44^, ^45^ ^]^ Figure 5h summarizes the energy consumption for serial and parallel programming of the conventional half voltage scheme and those of the suggested voltage scheme of Figure 5e. At the conventional half voltage scheme and serial programming, the total energy consumption was calculated to 0.51 J, which can be reduced to 0.31 J (39% reduction) by introducing the parallel programming scheme. At the suggested biasing scheme, the total energy consumption decreased drastically from 0.66 to 0.19 J (71% reduction). This energy reduction is attributed to the increased number of half‐selected cells (right panel of Figure 5d) biased to 1.3 V, suppressing the leakage current.

## 3 Conclusion

In this work, we proposed the Pt/Ta_2_O_5_/Nb_2_O_5‐_ *_x_*/Al_2_O_3‐_ *_y_*/Ti CTM device exhibiting self‐rectifying, nonlinear, highly uniform, analog, and low power operation characteristics. Moreover, the device retention was secured, making it practically available for the crossbar array applications. We proposed an electronic band diagram model supported by in‐depth spectroscopy analysis, which helped to understand the mechanism of the memristive system. We integrated the cell on a 32 × 32 crossbar array and suggested its energy‐efficient neuromorphic computing operation using a dedicated biasing scheme synchronized with the device's *I*–*V* characteristics. This high potential of the CTM system can be widely applicable for various applications where energy consumption is the matter, such as an edge neuromorphic device.

## 4 Experimental Section

### Fabrication of the Pt/Ta_2_O_5_/Nb_2_O_5‐_ *_x_*/Al_2_O_3‐_ *_y_*/Ti Device

For Pt (70 nm)/ Ta_2_O_5_ (10 nm)/ Nb_2_O_5‐_ *_x_* (28 nm)/ Al_2_O_3‐_ *_y_* (8 nm)/ Ti (50 nm) device integration, a 50‐nm‐thick Ti bottom electrode was evaporated and patterned by photolithography followed by a lift‐off process on SiO_2_/Si substrate. Then, the Al_2_O_3‐_ *_y_* tunneling layer was deposited by a thermal atomic layer deposition (ALD) at 250 °C using Trimethylaluminum and H_2_O as the Al precursor and oxygen source, respectively. The Nb_2_O_5‐_ *_x_* charge trap layer was sputtered at Ar and O_2_ mixed ambient using Nb metal target. Next, the Ta_2_O_5_ blocking layer was deposited using a PEALD at 225 °C using Tris(diethylamido)(tert‐butylimido)tantalum(V) and O_2_ plasma for the Ta precursor and the oxidant, respectively. Finally, the Pt top electrode was deposited by e‐beam evaporation and patterned using a lift‐off process. The line width was 5 µm, so the device area was 25 µm^2^ with a crossbar structure.

### Device Spectroscopy Analysis

The XPS depth profile and the cross‐section of the PTNAT sample were observed using XPS (Thermo VG, Sigma Probe) and scanning transmission electron microscopy (Talos F200X). For the XPS analysis, the Ar^+^ ion beam energy was set to 1 keV during the mono‐gun method etching. Each core level was checked with a 4 s time interval. The bandgap and electron affinity of the PTNAT sample was observed using REELS and UPS (Kratos, Axis‐Supra). For the analysis, the energy gap between valence band maximum and Fermi energy level was obtained from the low binding energy cutoff and the work function value from the high binding energy cutoff. The photon energy of light with the He UV emission was 21.22 eV for the UPS analysis, and each work function value was extracted from the gap between the photo energy and the high binding energy cutoff value.

### Electrical Measurements

The device was measured using a probe station and semiconductor parameter analyzer (Keithley 4200A‐SCS) for the single device and ArC ONE memristor characterization platform for the array device. During the electrical measurement, the Pt TE was biased while Ti BE was grounded.

## Conflict of Interest

The authors declare no conflict of interest.

## Supporting information

Supporting Information

## Data Availability

The data that support the findings of this study are available from the corresponding author upon reasonable request.

## References

[1] M. A. Zidan , J. P. Strachan , W. D. Lu , Nat. Electron. 2018, 1, 22.

[2] J. Zhou , K. Kim , W. Lu , IEEE Trans. Electron Devices 2014, 61, 1369.

[3] C. Hsu , I. Wang , C. Lo , M. Chiang , W. Jang , C. Lin , T. Hou , in 2013 Symposium on VLSI Technology, 2013, p. T166.

[4] J. H. Yoon , S. J. Song , I.‐H. Yoo , J. Y. Seok , K. J. Yoon , D. E. Kwon , T. H. Park , C. S. Hwang , Adv. Funct. Mater. 2014, 24, 5086.

[5] S. Gao , F. Zeng , F. Li , M. Wang , H. Mao , G. Wang , C. Song , F. Pan , Nanoscale 2015, 7, 6031. 25765948 10.1039/c4nr06406b

[6] J. H. Yoon , K. M. Kim , S. J. Song , J. Y. Seok , K. J. Yoon , D. E. Kwon , T. H. Park , Y. J. Kwon , X. Shao , C. S. Hwang , Adv. Mater. 2015, 27, 3811. 25973913 10.1002/adma.201501167

[7] K. M. Kim , J. Zhang , C. Graves , J. J. Yang , B. J. Choi , C. S. Hwang , Z. Li , R. S. Williams , Nano Lett. 2016, 16, 6724. 27661260 10.1021/acs.nanolett.6b01781

[8] J. H. Yoon , D. E. Kwon , Y. Kim , Y. J. Kwon , K. J. Yoon , T. H. Park , X. L. Shao , C. S. Hwang , Nanoscale 2017, 9, 11920. 28786468 10.1039/c7nr02215h

[9] Y. Kim , Y. J. Kwon , D. E. Kwon , K. J. Yoon , J. H. Yoon , S. Yoo , H. J. Kim , T. H. Park , J.‐W. Han , K. M. Kim , C. S. Hwang , Adv. Mater. 2018, 30, 1704320. 10.1002/adma.201704320 29318678

[10] Q. Luo , X. Zhang , Y. Hu , T. Gong , X. Xu , P. Yuan , H. Ma , D. Dong , H. Lv , S. Long , Q. Liu , M. Liu , IEEE Electron Device Lett. 2018, 39, 664.

[11] C. Li , Q. Xia , Handbook of Memristor Networks, Springer, Berlin 2019.

[12] S. Yu , B. Gao , Z. Fang , H. Yu , J. Kang , H.‐S. P. Wong , Adv. Mater. 2013, 25, 1774. 23355110 10.1002/adma.201203680

[13] C. S. Hwang , Adv. Electron. Mater. 2015, 1, 1400056.

[14] J. Sanghun , H. J. Hee , L. J. Hoon , C. Sangmoo , H. Hyunsang , K. Chungwoo , IEEE Trans. Electron. Dev. 2005, 52, 2654.

[15] J.‐S. Lee , J. Cho , C. Lee , I. Kim , J. Park , Y.‐M. Kim , H. Shin , J. Lee , F. Caruso , Nat. Nanotechnol. 2007, 2, 790. 18654433 10.1038/nnano.2007.380

[16] H.‐W. You , W.‐J. Cho , Appl. Phys. Lett. 2010, 96, 093506.

[17] K. M. Kim , B. J. Choi , M. H. Lee , G. H. Kim , S. J. Song , J. Y. Seok , J. H. Yoon , S. Han , C. S. Hwang , Nanotechnology 2011, 22, 254010. 21572205 10.1088/0957-4484/22/25/254010

[18] X. L. Shao , L. W. Zhou , K. J. Yoon , H. Jiang , J. S. Zhao , K. L. Zhang , S. Yoo , C. S. Hwang , Nanoscale 2015, 7, 11063. 26050964 10.1039/c4nr06417h

[19] J. Zhao , M. Zhang , S. Wan , Z. Yang , C. S. Hwang , ACS Appl. Mater. Interfaces 2018, 10, 1828. 29256591 10.1021/acsami.7b16214

[20] H. Schroeder , V. V. Zhirnov , R. K. Cavin , R. Waser , J. Appl. Phys. 2010, 107, 054517.

[21] A. Modelli , A. Visconti , R. Bez , in 2004 International Conference on Integrated Circuit and Design Technology, 2004, p. 211.

[22] T. R. Oldham , M. Friendlich , M. A. Carts , C. M. Seidleck , K. A. LaBel , IEEE Trans. Nucl. Sci. 2009, 6, 3280.

[23] R. J. Schwartz , Y. L. Chiou , H. W. Thompson , Thin Solid Films 1970, 6, 81.

[24] F.‐C. Chiu , Adv. Mater. Sci. Eng. 2014, 2014, 578168.

[25] H. C. Huang , T. E. Hsieh , J. Appl. Polym. Sci. 2010, 117, 1960.

[26] N. Fuschillo , B. Lalevic , N. Annamalai , W. Slusark Jr. , J. Non‐Cryst. Solids 1976, 22, 159.

[27] H. Birey , J. Appl. Phys. 1977, 48, 5209.

[28] V. A. Shvets , V. S. Aliev , D. V. Gritsenko , S. S. Shaimeev , E. V. Fedosenko , S. V. Rykhlitski , V. V. Atuchin , V. A. Gritsenko , V. M. Tapilin , H. Wong , J. Non‐Cryst. Solids 2008, 354, 3025.

[29] R. Simpson , R. G. White , J. F. Watts , M. A. Baker , Appl. Surf. Sci. 2017, 405, 79.

[30] B. R. King , H. C. Patel , D. A. Gulino , B. J. Tatarchuk , Thin Solid Films 1990, 192, 351.

[31] J. A. Rotole , P. M. A. Sherwood , Surf. Sci. Spectra 1998, 5, 18.

[32] J. C. Shank , M. B. Tellekamp , M. J. Wahila , S. Howard , A. S. Weidenbach , B. Zivasatienraj , L. F. J. Piper , W. A. Doolittle , Sci. Rep. 2018, 8, 12935. 30154545 10.1038/s41598-018-30727-9 PMC6113211

[33] M. Z. Kabir , S. Kasap , Handbook of Electronic and Photonic Materials, Springer, Berlin 2017.

[34] P. Chen , L. Gao , S. Yu , IEEE Trans. Multi‐Scale Comput. Syst. 2016, 2, 257.

[35] G. S. Kim , H. Song , Y. K. Lee , J. H. Kim , W. Kim , T. H. Park , H. J. Kim , K. M. Kim , C. S. Hwang , ACS Appl. Mater. Interfaces 2019, 11, 47063. 31741373 10.1021/acsami.9b16499

[36] H. Song , Y. S. Kim , J. Park , K. M. Kim , Adv. Electron. Mater. 2019, 5, 1800740.

[37] H. Song , J. An , S. Son , Y. S. Kim , J. Park , J. B. Jeon , G. Kim , K. M. Kim , Adv. Intell. Syst. 2020, 2, 2000014.

[38] Y. Lecun , L. Bottou , Y. Bengio , P. Haffner , Proc. IEEE 1998, 86, 2278.

[39] J. U. Kwon , Y. G. Song , J. E. Kim , S. Y. Chun , G. H. Kim , G. Noh , J. Y. Kwak , S. Hur , C.‐Y. Kang , D. S. Jeong , S. J. Oh , J. H. Yoon , ACS Appl. Mater. Interfaces 2022, 14, 44550. 36149315 10.1021/acsami.2c12247

[40] C. Li , D. Belkin , Y. Li , P. Yan , M. Hu , N. Ge , H. Jiang , E. Montgomery , P. Lin , Z. Wang , W. Song , J. P. Strachan , M. Barnell , Q. Wu , R. S. Williams , J. J. Yang , Q. Xia , Nat. Commun. 2018, 9, 2385. 29921923 10.1038/s41467-018-04484-2 PMC6008303

[41] Z. Wang , C. Li , P. Lin , M. Rao , Y. Nie , W. Song , Q. Qiu , Y. Li , P. Yan , J. P. Strachan , N. Ge , N. McDonald , Q. Wu , M. Hu , H. Wu , R. S. Williams , Q. Xia , J. J. Yang , Nat. Mach. Intell. 2019, 1, 434.

[42] R. Waser , R. Dittmann , G. Staikov , K. Szot , Adv. Mater. 2009, 21, 2632. 10.1002/adma.200900375 36751064

[43] Y. Zhang , G.‐Q. Mao , X. Zhao , Y. Li , M. Zhang , Z. Wu , W. Wu , H. Sun , Y. Guo , L. Wang , X. Zhang , Q. Liu , H. Lv , K.‐H. Xue , G. Xu , X. Miao , S. Long , M. Liu , Nat. Commun. 2021, 12, 7232. 34903752 10.1038/s41467-021-27575-z PMC8668918

[44] Z.‐Y. Shao , H.‐M. Huang , X. Guo , Solid State Ionics 2021, 370, 115746.

[45] J. Woo , K. Moon , J. Song , S. Lee , M. Kwak , J. Park , H. Hwang , IEEE Electron Device Lett. 2016, 37, 994.

