# Supplementary material for: Retention Secured Nonlinear and Self‐Rectifying Analog Charge Trap Memristor for Energy‐Efficient Neuromorphic Hardware
Source: Adv Sci (Weinh). 2022 Nov 27;10(3):2205654. doi: 10.1002/advs.202205654 (PMC9875615; doi:10.1002/advs.202205654)
Supplement: Supplementary file 1 — Supporting Information [file ADVS-10-2205654-s001.pdf]

ADVANCED SCIENCE
Open Access

Supporting Information

for *Adv. Sci.*, DOI 10.1002/advs.202205654

Retention Secured Nonlinear and Self-Rectifying Analog Charge Trap Memristor for Energy-Efficient Neuromorphic Hardware

*Geunyoung Kim, Seoil Son, Hanchan Song, Jae Bum Jeon, Jiyun Lee, Woon Hyung Cheong, Shinhyun Choi and Kyung Min Kim**

Supporting Information

Retention secured nonlinear and self-rectifying analog charge trap memristor for energy-efficient neuromorphic hardware

*Geunyoung Kim, Seoil Son, Hanchan Song, Jae Bum Jeon, Jiyun Lee, Woon Hyung Cheong, Shinhyun Choi, and Kyung Min Kim**

This PDF file includes:

Supporting Figures

Supporting Figures

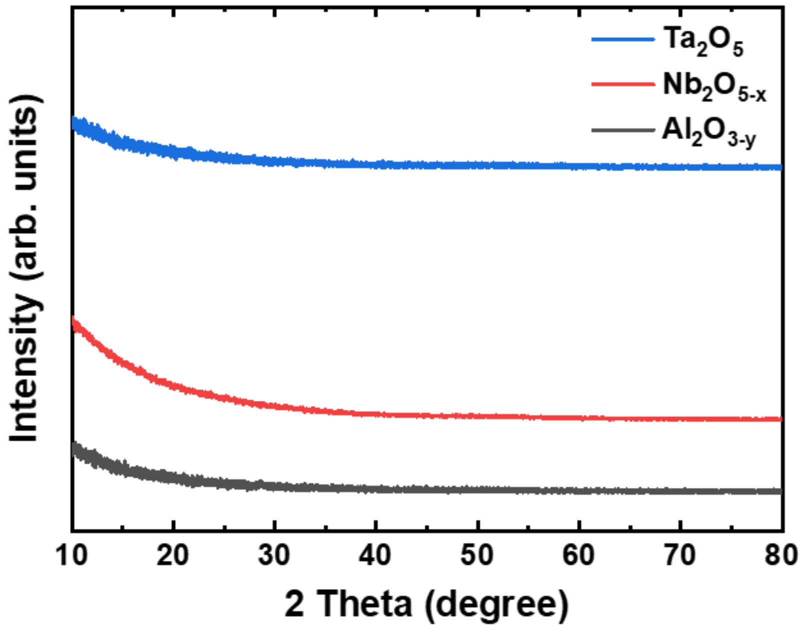

Figure S1. XRD data of PTNAT device. The x-ray diffraction results of Ta_2O_5, Nb_2O_{5-x} and Al_2O_{3-y} films on a SiO_2 substrate. All thin films were amorphous as deposited.

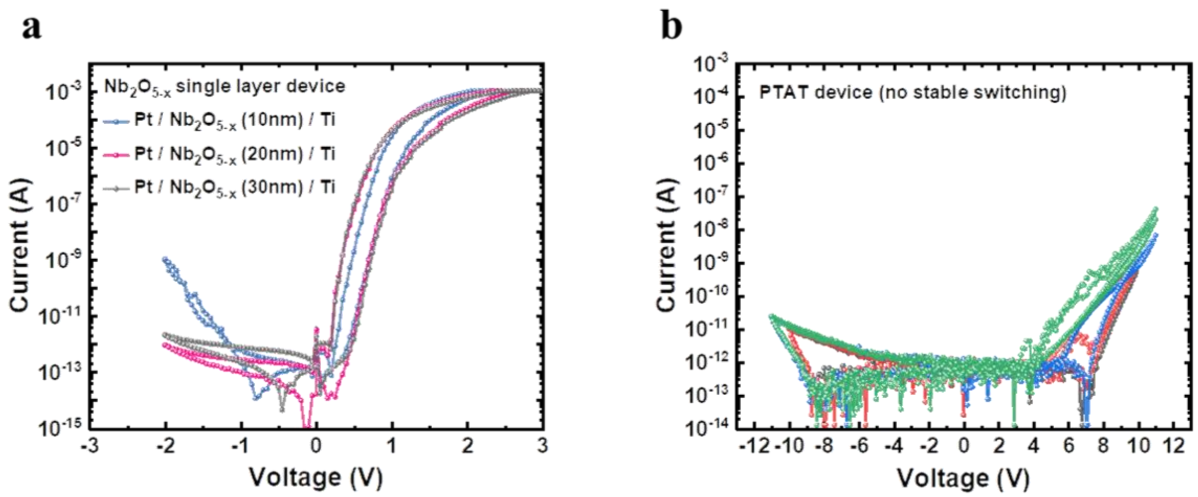

Figure S2. PNT & PTAT device memory characteristics. a) Pt/Nb_2O_{5-x}(10nm, 20nm, 30nm)/Ti (PNT) single layer device I-V curves. The device showed memory operation only with the single Nb_2O_{5-x} layer. b) Pt/Ta_2O_5/Al_2O_{3-y}/Ti (PTAT) double layer device I-V curves. The device showed no stable memory operation.

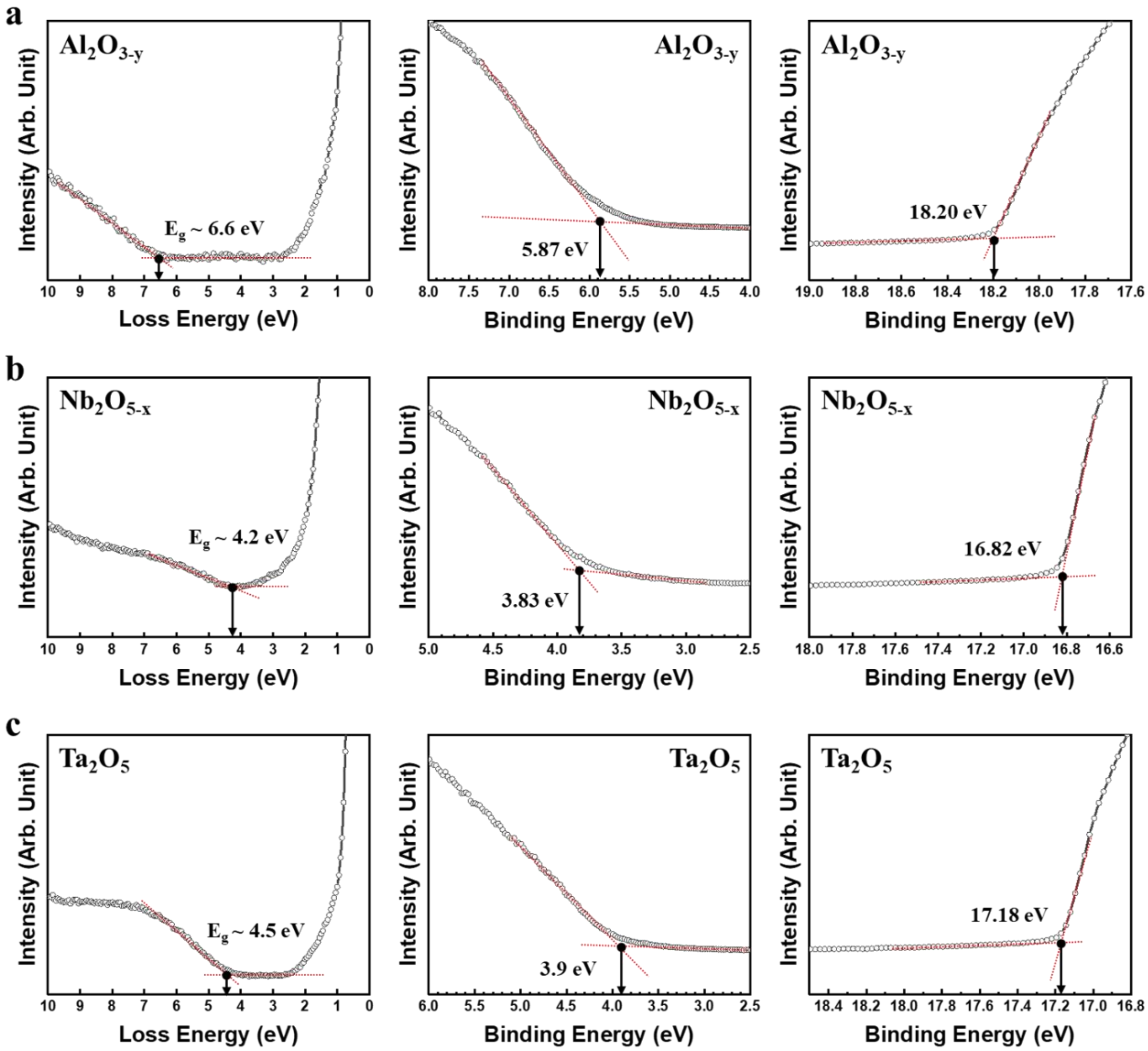

d

REELS & UPS results		
Al_2O_{3-y}	E_g	6.6 eV
	χ	2.29 eV
Nb_2O_{5-x}	E_g	4.2 eV
	χ	4.03 eV
Ta_2O_5	E_g	4.5 eV
	χ	3.44 eV

Figure S3. REELS & UPS analysis results. REELS and UPS binding energy cutoff results of a) Al_2O_{3-y}, b) Nb_2O_{5-x}, and c) Ta_2O_5. Used equation for the χ value extraction is as follows:

$$\varphi = h\omega - E_{high\ cutoff} \quad (1)$$

$$\chi = \varphi - (E_g - E_{low\ cutoff}) \quad (2)$$

where φ is the work function, $h\omega$ is the photo energy of light (He UV emission at 21.22 eV), and E_{cutoff} values were obtained by fitting the energy graph of the UPS at high and low binding energies. d) Calculated E_g and χ values of each oxide layer.

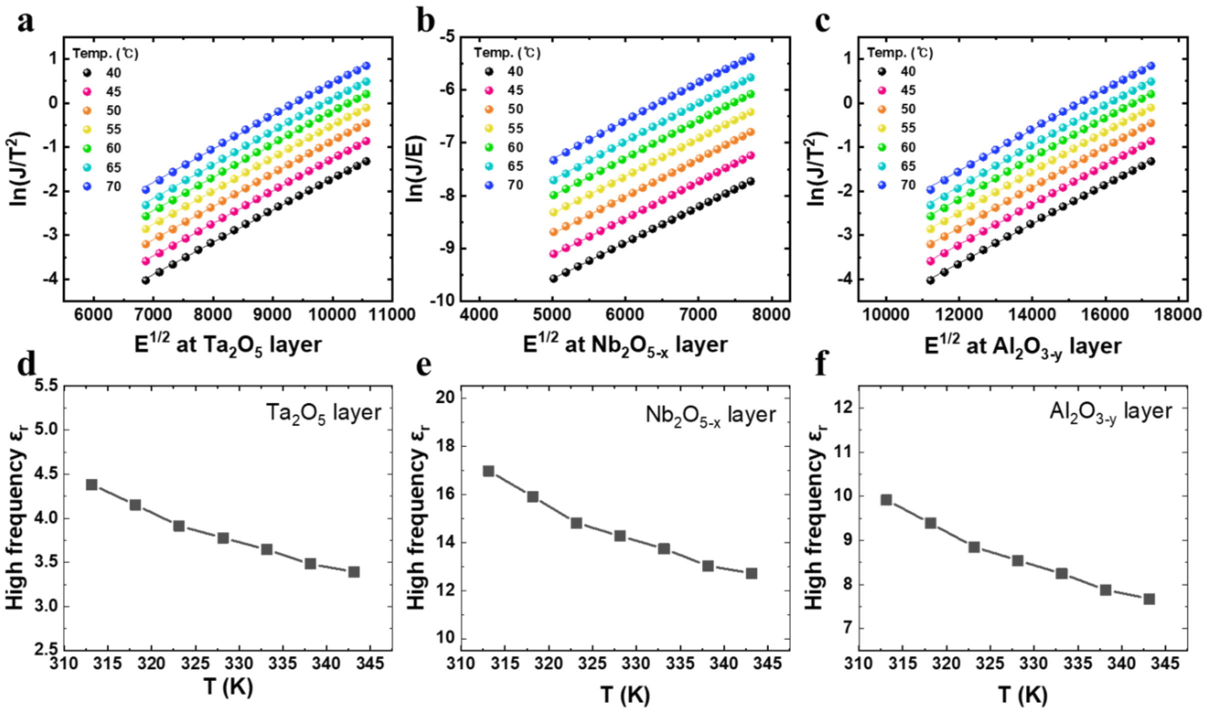

Figure S4. Conduction mechanism fitting results limited by different oxide layers. Fitting results of a) Schottky emission with Ta_2O_5 partial field, b) Poole-Frenkel (P-F) emission with Nb_2O_{5-x} partial field and c) Schottky emission with Al_2O_{3-y} partial field calculation. Due to the high affinity value of Nb_2O_{5-x} layer ($\chi \approx 4$), bulk limited mechanism (temperature dependent P-F emission) was assumed. The calculated ε_{op} values of each dielectric layers were d) 3.39 to 4.38 for Ta_2O_5 layer, e) 12.72 to 16.97 for Nb_2O_{5-x} layer, and f) 7.68 to 9.92 for Al_2O_{3-y} layer with LRS fitting. Compare with the reference values, the fitted results were not well matched with Nb_2O_{5-x} ($\varepsilon_{op} \approx 5$) and Al_2O_{3-y} ($\varepsilon_{op} \approx 2.9$) layers.

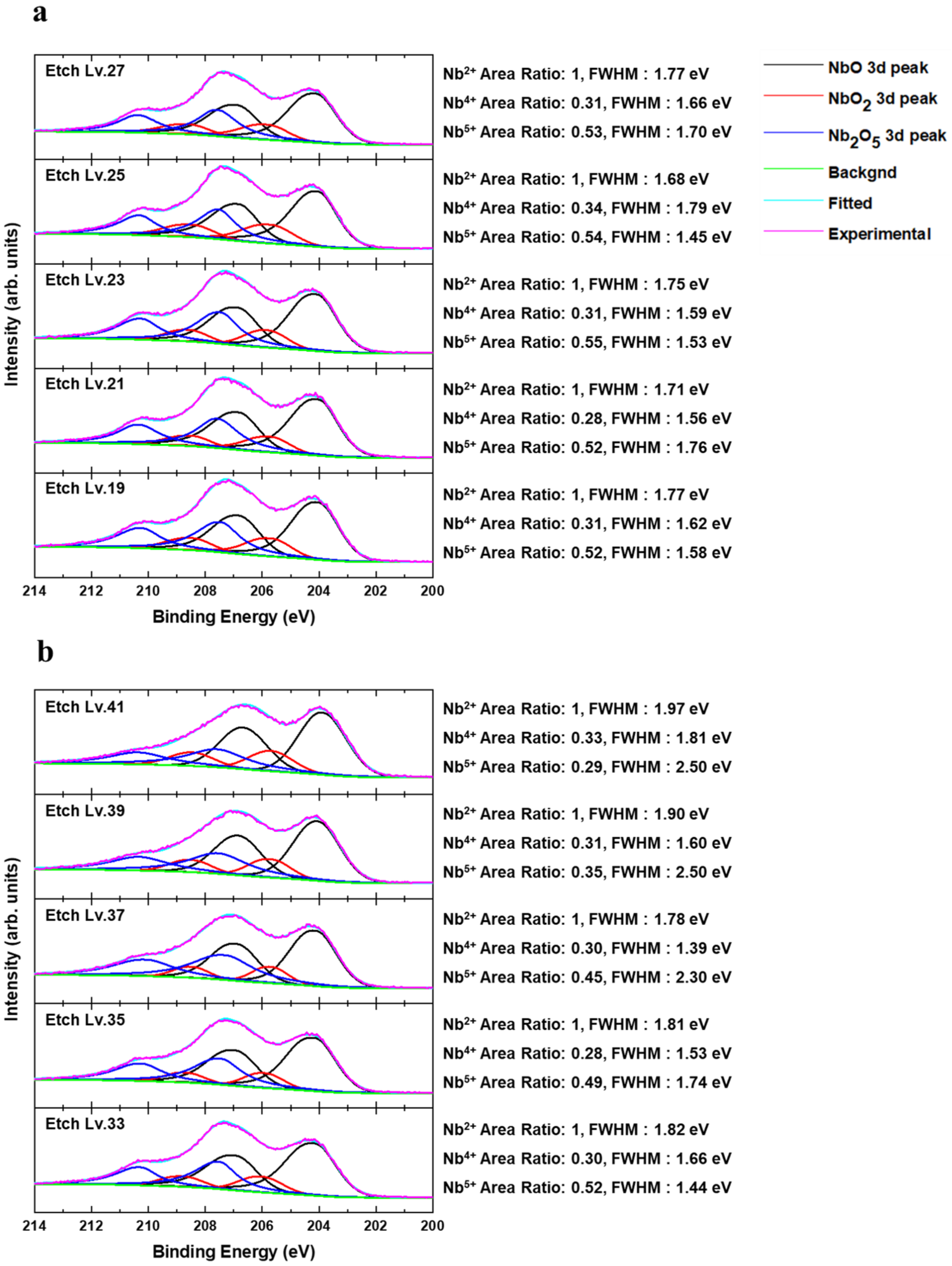

Figure S5. Nb 3*d* XPS spectra deconvolution results of PTNAT device. Nb 3*d* XPS depth profile from a) Nb_2O_{5-x} bulk (19~27 etch level) to b) Nb_2O_{5-x}/Al_2O_{3-y} interface region (33~41 etch level). In the bulk region, analysis results show various sub-phases are composed. As the etch level increased near to Nb_2O_{5-x}/Al_2O_{3-y} interface region, binding energy shift occured driven from the additional defects.

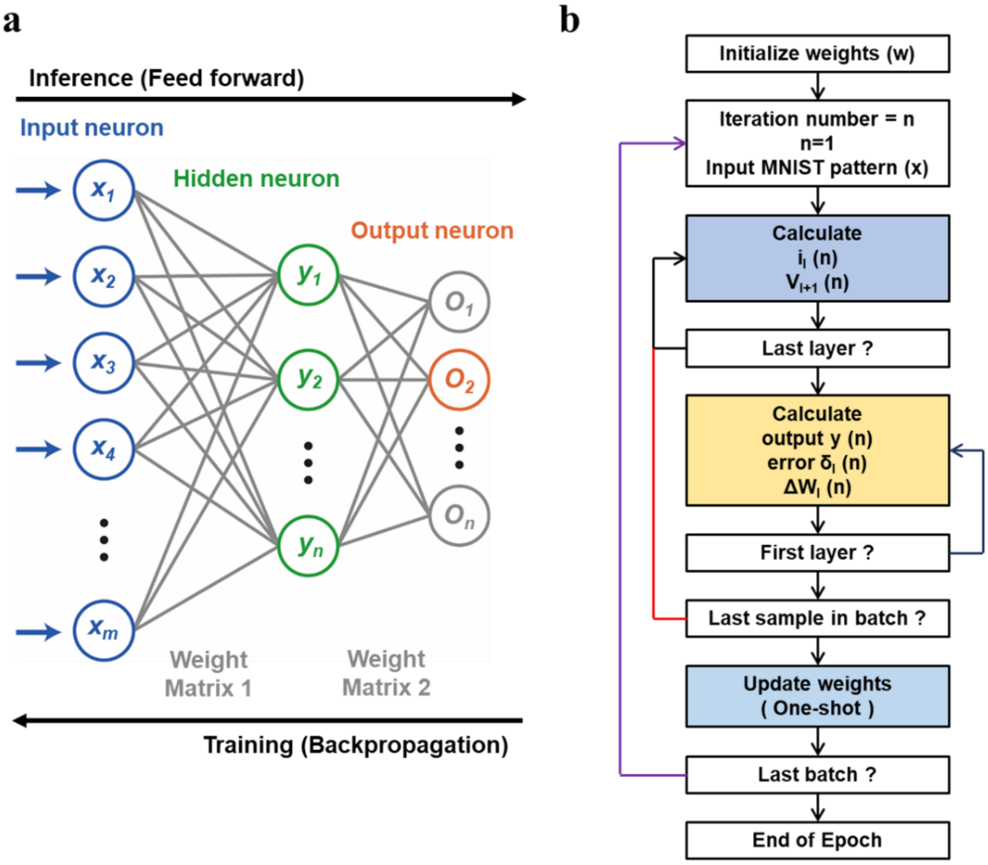

Figure S6. Memristive neural network simulation for MNIST classification. a) Schematic diagram of double-layer perceptron neural network. b) Flow chart of the training process. After the backpropagation, output value (y), error value (δ), and desired weight update value (ΔW) were calculated. A single programming pulse based on the synaptic property was applied to the selected cell for the weight update.

a

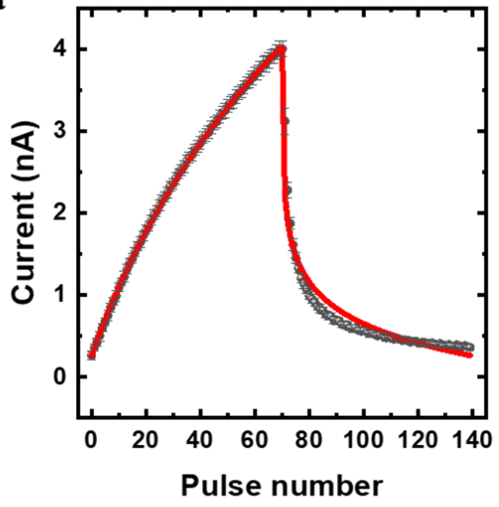

b

Fitting parameter		
Potentiation	α_p	0.85
	β_p	8.56×10^{-11}
Depression	α_d	8.05
	β_d	2.40×10^{-8}
G Bound	G_{max}	4.00×10^{-9}
	G_{min}	2.64×10^{-10}
Variability	σ_{bound}	0.08
	σ_{slope}	0.07
	$\sigma_{temporal}$	0.05

Figure S7. MNIST recognition test fitting parameter of PTNAT device. a) Synaptic plasticity characteristic of PTNAT device. b) Fitting parameter from the synaptic plasticity result. Used equation for the fitting is as follows.

$$G(n+\Delta n) = G(n) + \frac{G_{max}-G_{min}}{\alpha_p} ln(1 + \frac{\alpha_p}{G_{max}-G_{min}} \beta_p \Delta n\, exp\left(-\alpha_p \frac{G(n)-G_{min}}{G_{max}-G_{min}}\right)) \quad (3)$$

$$G(n+\Delta n) = G(n) - \frac{G_{max}-G_{min}}{\alpha_d} ln(1 + \frac{\alpha_d}{G_{max}-G_{min}} \beta_d \Delta n\, exp\left(-\alpha_d \frac{G_{max}-G(n)}{G_{max}-G_{min}}\right)) \quad (4)$$

$$\beta_{p,d} = \frac{G_{max}-G_{min}}{\Delta n_{max}} \frac{exp(\alpha_{p,d})-1}{\alpha_{p,d}} \quad (5)$$
